# Steviol Glycosides from *Stevia rebaudiana*: An Updated Overview of Their Sweetening Activity, Pharmacological Properties, and Safety Aspects

**DOI:** 10.3390/molecules28031258

**Published:** 2023-01-27

**Authors:** Adriana Monserrath Orellana-Paucar

**Affiliations:** 1 Nutrition and Dietetics School, Faculty of Medical Sciences, University of Cuenca, Cuenca 010204, Ecuador; adriana.orellanap@ucuenca.edu.ec; 2 Pharmacology and Nutritional Sciences Interdisciplinary Research Group, Faculty of Medical Sciences, University of Cuenca, Cuenca 010204, Ecuador

**Keywords:** *Stevia rebaudiana*, stevia, steviol glycosides, sweetener, bioactivity, safety, toxicity

## Abstract

This literature-based review synthesizes the available scientific information about steviol glycosides as natural sweeteners and molecules with therapeutic potential. In addition, it discusses the safety concerns regarding human consumption. Steviol glycosides exhibit a superior sweetener proficiency to that of sucrose and are noncaloric, noncariogenic, and nonfermentative. Scientific evidence encourages stevioside and rebaudioside A as sweetener alternatives to sucrose and supports their use based on their absences of harmful effects on human health. Moreover, these active compounds isolated from *Stevia rebaudiana* possess interesting medicinal activities, including antidiabetic, antihypertensive, anti-inflammatory, antioxidant, anticancer, and antidiarrheal activity. The described bioactivities of steviol glycosides deserve special attention based on their dose dependence and specific pathological situations. Further clinical research is needed to understand underlying mechanisms of action, therapeutic indexes, and pharmacological applications.

## 1. Introduction

*Stevia rebaudiana* Bertoni (Asteraceae) is a small perennial herb endemic to South America. Dry stevia leaves have been used to sweeten traditional bitter drinks such as mate tea [1]. Due to its sweetening and pharmacological qualities, stevia has attracted international commercial attention and scientific interest. Japan was the first nation outside of Latin America to cultivate and market stevia as a sucrose alternative. China, Malaysia, Singapore, South Korea, Taiwan, and Thailand are also merchandising it. Stevia plantations can now be found in Southeast Asia, the U.S., Canada, and Europe [2].

### 1.1. Chemical Structure of Steviol Glycosides

**Figure 1:**
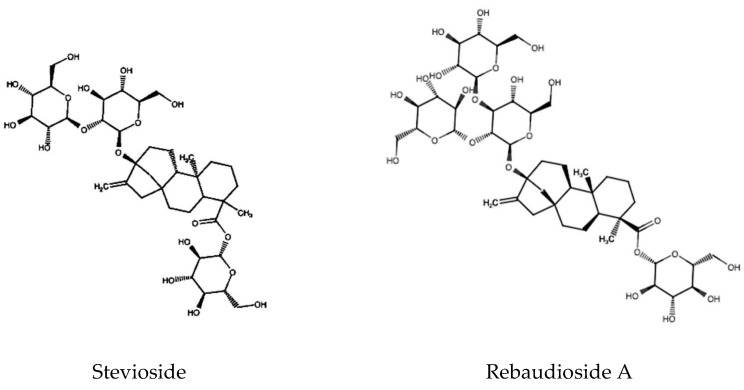
Structure of the main steviol glycosides in *S. rebaudiana*.

Only 18 of the 150–300 species from the genus Stevia exhibit sweetening properties. Among them, *Stevia rebaudiana* is the sweetest variety. The eight steviol glycosides (ent-kaurene glycosides), including dulcoside A, rebaudiosides A-E, steviolbioside, and stevioside, naturally occur in its leaves. Their total content oscillates from 4% to 20% depending on genotype and culture conditions. The two major sweeteners are stevioside (~9.1%) and rebaudioside A (~3.8%) [3,4] (Figure 1).

### 1.2. Pharmacokinetics

**Figure 2:**
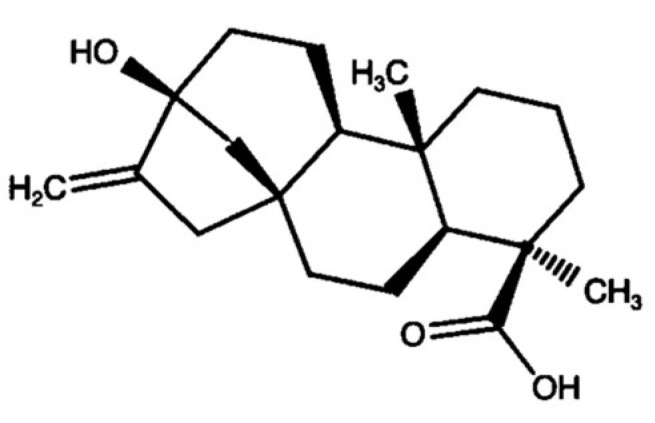
Structure of steviol, the common metabolite of steviol glycosides.

Preclinical and clinical studies have revealed that neither digestive enzymes nor gastric juice can decompose stevioside [5,6,7]. In addition, oral stevioside seems not to be absorbed at the upper-small-intestine level, probably due to its high molecular weight [7]. In the lower gastrointestinal tracts of rats, mice, pigs, and humans, stevioside can be degraded by bacterial intestinal flora (Bacteroides genus), transforming it into free steviol, an aglycone of steviol glycosides [5,6,7,8,9,10]. A study of human volunteers showed no detectable amount of stevioside, free steviol, or any other steviol metabolite in the blood after stevioside consumption (750 mg/day). Nevertheless, steviol was present in the feces [11] (Figure 2). 

In rats, steviol was detected in a portal venous blood sample after oral administration of stevioside [7]. Experiments in everted rat intestinal sacs revealed a much more active transport for steviol than for stevioside. Similarly, research on the human intestinal Caco-2 cell line evidenced deficient transport of stevioside in contrast to steviol [10]. Hence, steviol, not stevioside, is the compound absorbed after oral administration. 

After a single oral dose of radioactive ^3^H-stevioside (125 mg/kg) in rats, a maximum blood radioactivity level of 4.8 µg/mL with an elimination half-life of 24 h was found eight hours after administration [12]. After intravenous administration, the highest accumulation of radioactive 131I-stevioside in rats was detected in the liver (52% of injected dose). HPLC analyses of bile revealed steviol as the primary metabolite among other, unknown compounds [13]. Conversion of stevioside and rebaudioside A into steviol occurs in the liver (phase I metabolism) and is excreted through urine [14,15]. Steviol undergoes enterohepatic reabsorption and is conjugated into steviol glucuronide (phase II metabolism). This major metabolite is rapidly excreted from the body [14]. 

For humans and rats, steviol glucuronide is the standard blood metabolite. Nevertheless, its excretion routes are distinct due to a difference in molecular weight threshold for organic anions eliminated through the bile. For humans, organic ions with a molecular weight superior to 600 Da, and for mice, those weighing more than 325 Da are excreted through bile instead of urine. Steviol glucuronide (512.9 Da) is excreted via human kidneys and through bile in rats [14]. Experimental studies that investigated renal elimination detected nephrotoxicity in rats after subcutaneous administration of stevioside 1.5 g/kg (equivalent to 250 times the average daily human consumption). Increased urinary glucose and plasma creatinine levels were reported after stevioside administration [16]. Thus, stevioside apparently interferes with secretory transport systems at very high doses. Further studies of secretory-transport-system inhibition are needed in order to employ stevioside to delay drug clearance and enhance these systems’ efficacy in the human body. Stevioside can be considered safe when consumed according to the acceptable daily intake (ADI). 

## 2. Steviol Glycosides as Noncaloric Sucrose Replacements and Noncariogenic Sweeteners

To date, the non-nutritive sweeteners aspartame, acesulfame potassium, and saccharine have been commonly used to replace sucrose for medical problems such as hypertension, type 2 diabetes, and obesity. Nevertheless, there is a permanent concern about their adverse effects, especially regarding neurological consequences, carcinogenesis, appetite increase, and others [17,18,19]. Hence, increased demand for novel, safe, noncaloric, and noncariogenic natural sweeteners is arising. 

Stevia has been traditionally employed as a sweetener in South America. Currently, this use is spread all over the world. In Brazil, Korea, and Japan, stevia extract and stevioside are officially accepted as food additives. Stevia entered Japan’s market in 1970, when artificial sweeteners were banned. In the U.S., stevia leaves and their extract were approved in 2007 as a ‘dietary supplement’ for the Joint FAO/WHO Expert Committee on Food Additives (JECFA) [20]. In 2011, the European Union certified steviol glycosides (SGs) as a food additive, whereas in 2018, the FDA conferred the ‘Generally Recognized As Safe’ (GRAS) status to stevia leaf extract [21]. The European Food Safety Authority (EFSA) established the acceptable daily intake (ADI) of 4 mg/kg/day for steviol glycosides. Noteworthily, there is clinical evidence of no pharmacological effects of SGs when they are administered as sweeteners, probably due to the minor amount necessary to consume for this purpose [22].

Rebaudioside A is around 450-fold and stevioside 300-fold sweeter than sucrose (0.4% solution) [23]. Along with their sweetness, stevioside and rebaudioside A have some bitterness and undesirable metallic aftertaste: stevioside more than rebaudioside A [24]. In ice cream, the aftertastes of rebaudiosides D and M were perceived as sweeter, creamier, milkier, and more agreeable than was that of rebaudioside A. The sensory perception attributed to rebaudiosides D and M was similar to that of sucrose [25]. Nevertheless, rebaudiosides D and M were still negatively perceived as artificial [26]. This palatable problem may be successfully controlled through addition of monosaccharide residues into the SG structure via enzymatic glycosylation [27]. Suitable sensory quality and appropriate physicochemical features were attributed to apple preserves that contained SGs instead of glucose [28]. Likewise, replacing 25% of sucrose with SGs in bakery products led to improved texture, color, browning index, and sensory perception [29,30]. 

In addition to their high sweetening capability, safety, and noncaloric nature, in vivo and in vitro studies have demonstrated the noncariogenic properties of stevia extract, stevioside, and rebaudioside A [31,32]. Dental caries is a highly prevalent disease that affects around 30–60% of children worldwide [33]. Dental hard tissues are affected by biofilm and plaque development that involve surface roughness, demineralization, cavitation with pulp compromise, swelling, and abscess, leading to systemic impact due to infection spreading through the jawbone. Some factors that promote caries formation are poor oral hygiene habits, eating habits (i.e., high consumption of simple carbohydrates), genetic susceptibility, and depleted oral concentration of calcium and phosphate ions, among others. *Streptococcus mutans* and *Lactobacillus acidophilus*, from oral microbiota, are acid-producing bacteria. This acid’s reaction with fermentable carbohydrates (mainly sucrose) causes erosion of superficial tooth enamel. Salivary calcium and phosphate ions remineralize the tooth, occasionally producing calcification. Nevertheless, this extended acid contact generates subsurface demineralization, shaping a cavity [34]. 

Thus, the nonfermentative and noncariogenic properties of stevioside and rebaudioside A were demonstrated in preclinical models. Rats fed with a 30% sucrose-added diet displayed increased *Streptococcus sobrinus* counts (*p* < 0.05) and sulcal caries scores (*p* < 0.02) from the groups supplemented with 0.5% stevioside and 0.5% rebaudioside A and the control group without additives. The difference in the tested concentrations of the sweeteners was correlated to their sweetening capacities and the corresponding ADIs. No differences were observed among these last three animal sets [35]. This finding is consistent with the reported capability of stevioside to control *Streptococcus mutans* acid production and growth [36,37]. Other studies are needed to clinically evaluate the anticariogenic properties of SGs in the long term and the presence of other sweeteners. 

## 3. Pharmacological Properties of Steviol Glycosides

### 3.1. Antidiabetic Action

Diabetes is a chronic metabolic disease characterized by an insufficient amount of insulin due to pancreatic beta cell destruction (type 1 diabetes) or cell inability to effectively use insulin due to impaired responsiveness (type 2 diabetes) [38]. This disease affects around 536.6 million people worldwide [39]. Among the factors that contribute to type 2 diabetes mellitus (T2DM) are unhealthy dietary habits, poor physical activity, and genetic predisposition. In T2DM, glucose metabolism in the liver is impaired, and its peripheral tissues resist average insulin concentrations. Moreover, atherosclerosis development and cardiovascular disorders are the most common T2DM complications [40]. 

A study with and without stevioside was performed in type 2 diabetic Goto–Kakizaki (GK) and normal Wistar rats to better understand the hypoglycemic activity of stevioside [41]. In diabetic rats, stevioside (0.2 g/kg; i.v. administration) decreased glucose blood levels and increased insulin responses and reactions to an intravenous glucose tolerance test (IVGT). On the other hand, stevioside enhanced insulin levels above basal during the IVGT, without altering blood glucose response, in normal rats. This research suggested stevioside as a potential drug candidate to treat type 2 diabetes. This finding correlates with the capability of stevioside to exert dose-dependent hypoglycemic activity and reduce insulin resistance in diabetic streptozocin (STZ)-induced rats. Regarding the potential mechanisms of action, it is implied that stevioside stimulates insulin secretion and increases insulin sensitivity due to gluconeogenesis retardation caused by a decrease in phosphoenol pyruvate carboxy kinase (PEPCK) gene expression in the rat liver [42]. 

Moreover, rebaudioside A increased insulin production in isolated murine islets of Langerhans depending on extracellular Ca^2+^ concentration [43]. Stevioside and rebaudioside A acted as receptor ligands, mimicking the insulin effect. SGs increased the glucose intake in rat fibroblasts [44]. Similar to insulin action, an increase in glucose transport activity was triggered by SGs in HL-60 human leukemia and SH-SY5Y human neuroblastoma cells. Moreover, SGs and insulin promoted PI3K and Akt phosphorylation. Thus, it was implied that GLUT translocation is related to PI3K/Akt pathway modulation [45]. Evidence shows that stevioside can increase insulin-mediated glucose transport into skeletal muscle and insulin sensitivity in insulin-resistant and insulin-sensitive rats [46].

In addition, stevioside and rebaudioside A increased the activity of TRPM5, a Ca^2+^-activated cation channel in pancreatic β-cells. Consequently, the insulin secretion associated with TRPM5 increased and prevented hyperglycemia in high fat diet induced diabetes mice [47]. In vivo and ex vivo studies in rats demonstrated that neither dietary supplementation with stevioside nor that with rebaudioside A (500 and 2500 mg/kg) altered blood glucose, insulin, or the insulin resistance index. Nevertheless, SGs normalized lipid metabolism and protected internal organs from damage in this model [48]. 

Potential undesired effects on metabolism are negligible, since SGs appear not to act as glucocorticoid receptor (GR) agonists and therefore do not hamper expression of GR-target genes, GR protein levels, or GRs on peripheral blood mononuclear cells [49]. Regarding normal glucose blood levels, an oral administration of 5.5 mg/kg/day of stevioside for 15 days in normal rats caused no effect with stevioside. In contrast, with stevia (20 mg/kg/day), plasma glucose concentration lowered under basal levels due to decreased activity of pyruvate carboxylase and PEPCK [50]. Stevioside does not exert an antihyperglycemic effect at normal glucose concentrations, but it does so at high blood glucose levels in diabetic rats. On the other hand, the observed hypoglycemic effect of stevia could be explained with the presence of another constituent in the extract, different from stevioside, without its selectivity. Clinical evidence supports long-term consumption of stevioside (250 mg, three times/day for three months) with no effect on normal glucose concentration levels or blood pressure [11].

### 3.2. Antihypertensive Activity

Around 8.5 million deaths associated with a systolic blood pressure >115 mmHg were reported globally. Nearby 88% of this quantity corresponds to low- and middle-income countries [51]. Accordingly, the estimation of hypertension prevalence is higher in low- and middle-income countries (1.04 billion) than in high-income countries (349 million) [52]. Among the risk factors for hypertension are obesity, alcohol consumption, poor physical activity, high sodium intake, and unhealthy dietary habits. Uncontrolled hypertension can lead to cardiovascular and kidney disorders. Since treatment of hypertension is long-term, there is a particular interest in the search for effective therapeutic options with minimal or no adverse effects. In this context, there is evidence of stevioside and rebaudioside A inducing vasodilation, diuresis, and natriuresis with a decrease in plasma volume, leading to general reductions in arterial pressure in preclinical and clinical assessments [53,54]. The hypotensive effect observed in rats after chronic oral administration (30 days) of 2.67 g stevia leaves/day was confirmed in spontaneously hypertensive rats. In that murine model, stevioside (100 mg/kg; i.v.) was able to reduce blood pressure with no change in serum epinephrine, norepinephrine, or dopamine levels [55]. 

These findings coincide with the effects observed in human patients with mild to moderate hypertension. After one year of continued consumption of 750 mg/day of stevioside and after two years of daily ingestion of 1500 mg/day of stevioside, a significant decrease in systolic and diastolic blood pressure was observed without modifications in blood biochemistry values or the left ventricular mass index [54]. In addition, intraperitoneal stevioside caused a dose-dependent relaxation of vasopressin-induced vasoconstriction in isolated aortic rings and failed to inhibit it in a Ca^2+^-free medium. This result suggests that stevioside triggers vasorelaxation via inhibition of Ca^2+^ reflux into the blood vessel [56]. 

Noteworthily, the antihypertensive effect of stevioside requires relatively higher doses than the acceptable daily intake (ADI). There is no evidence of a hypotensive effect in humans with normal arterial pressure levels [11]. Therefore, stevioside is an attractive candidate for further investigation due to its selective antihypertensive effect. 

### 3.3. Anti-Inflammatory Property

Chronic inflammation is characterized by continual recruitment of monocytes and lymphocytes, as well as tissue damage because of a persistent stimulus. Numerous chronic diseases, comprising autoimmune conditions, and metabolic disorders, including atherosclerosis, obesity, fibrosis, and cancer, are primarily influenced by chronic inflammation [57]. Proinflammatory cytokines play a role in stimulating inflammatory responses and are generated mainly from activated macrophages. Interesting results were observed when the releases of proinflammatory cytokines (TNF-α and IL-1β) and nitric oxide were measured in a human monocytic THP1 cell line. In lipopolysaccharide (LPS)-stimulated THP1 cells, stevioside (1mM) inhibited NF-κB. This transcription factor controls expression of inflammatory cytokines; in non-LPS-stimulated THP1 cells, the same concentration of stevioside promoted their release moderately [58]. After a 7-day assessment of the capability of stevioside (10 mg/kg/day) to regenerate muscular tissue following cardiotoxin-induced injury in Wistar rats, stevioside did not boost muscle regeneration but enhanced satellite cell activation through modulation of the NF-κB signaling pathway, increasing the number of myonuclei [59]. 

Moreover, stevioside prevented in vitro upregulation of genes involved in liver inflammation. In silico assays demonstrated its antagonistic action in two proinflammatory receptors: tumor necrosis factor receptor (TNFR)-1 and Toll-like receptor (TLR)-4-MD2. Stevioside appears to also be beneficial for healthy people, as an enhancer of the innate immune system [58]. In addition to stevioside, steviol was found responsible for inhibiting TPA-induced inflammation [60]. Thus, steviol and stevioside may be helpful as dietary supplementation for supporting muscle recovery and could be suggested as good candidates to be further developed as new drugs to treat inflammation. 

### 3.4. Antioxidant Activity

Increased production of reactive oxygen/nitrogen species overcomes the antioxidative defenses of the body under oxidative stress, which causes tissue damage, accelerated cell death, and oxidative modification of biological macromolecules. Hence, oxidative stress is the pathological state at the root of many diseases [61].

Liver injury induced via thioacetamide decreases its antioxidant capability through downregulation of nuclear erythroid factor 2 (Nrf2). Stevioside coadministration (20 mg/kg, twice a day) upregulated Nrf2 levels in murine models, and, accordingly, no elevation of oxidative markers was observed [62]. Likewise, a combination of stevioside, rebaudioside A, rebaudioside C, and dulcoside A enhanced the viability of rat cardiac fibroblasts when exposed to hydrogen peroxide, as well as augmenting the concentration and activity of catalase and superoxide dismutase [44]. The antioxidant effect of stevioside and rebaudioside A was confirmed in a fish model. Both effectively controlled lipoperoxidation and protein carbonylation [63]. Furthermore, stevioside prevented oxidative DNA damage in the livers and kidneys of a type 2 diabetes murine model. The in silico results thereof revealed the stevioside’s potential mechanism of action associated with its ability to inhibit beta-adrenergic and G-protein-coupled receptor kinases [64].

In addition, antioxidant properties were evaluated in food applications. SGs (50, 125, and 200 mg/L) decreased degradation rates of antioxidants (ascorbic and dehydroascorbic acid) in a dose-dependent manner. Higher concentrations of acids and sweeteners displayed more potent antioxidant action. Interestingly, no variation in SG concentration was observed [65]. Similarly, preservation of fruit beverages with stevia showed improved antioxidant indexes and increased sweetness [66].

### 3.5. Anticancer Action

According to the American Cancer Society, there will be around 19 million cancer survivors in 2024. The three most common types of cancer in men are prostate (43%), colon (9%), and melanoma (8%), and in women, they are breast (41%), uterine (8%), and colon cancer (8%) [67]. Lung (1.35 million cases), breast (1.15 million), and colorectal (1 million) cancers are the three most frequently diagnosed types. Lung (1.18 million cases), stomach (700,000 cases), and liver cancers (598,000 deaths) are the three most frequently fatal types [68]. Cancer therapy’s primary limitations are toxicity, poor tolerability, and adherence [69]. Therefore, novel drug treatments with no minimum adverse effects are urgently required. 

An experimental study in vitro showed that stevioside, steviol, and isosteviol could inhibit the carcinogenic effects induced via Epstein–Barr virus early antigen (EBV-EA) activation mediated through 7,12-dimethylbenz[a]anthracene (DMBA) and TPA in mouse skin [70]. In vivo studies confirmed this finding. The activity of the well-known tumor promoter, 12-O-tetradecanoylphorbol-13-acetate (TPA), was successfully inhibited with stevioside in a murine skin-cancer model [71]. In addition, stevioside reduced mammary adenoma incidence in F344 rats [72]. 

Stevioside and steviol decreased the viability of human colon carcinoma cells. Steviol inhibited DNA synthesis and induced mitochondrial apoptosis [73]. In vitro analysis also showed potential activity of steviol glycosides against breast cancer cells [74]. Furthermore, steviol inhibited human gastrointestinal cancer cell growth through caspase-3 activation and mitochondrial apoptotic pathway triggering, and it increased the Bax/Bcl-2 ratio and stimulated p21 and p53 expression. Its pharmacological activity was comparable with that of 5-fluorouracil. Noteworthily, the cytotoxicity exerted by steviol against cancer cells was higher than the action displayed in normal cells [75]. Regarding mechanism of action, stevioside and steviol were able to inhibit two cancer pharmacotherapy targets: DNA polymerases and human DNA topoisomerase II [60]. Altogether, these results suggest stevioside, steviol, and isosteviol as valuable chemotherapy candidates to be further investigated for cancer therapy.

### 3.6. Antidiarrheal Activity

Bacterial enterotoxins increase intestinal-fluid and chloride-ion hypersecretion, leading to dehydration and electrolyte imbalance. This anion secretion is mediated with the cystic fibrosis transmembrane conductance regulator (CFTR), a critical cAMP-activated chloride channel. Steviol and dyhidrosteviol inhibit the CFTR [23]. Moreover, stevioside controls intestinal smooth muscle contraction [76]. 

These properties and the fact that SGs may possess antibacterial and antiviral activity suggest SGs as a potential treatment for diarrhea. SGs have demonstrated antibacterial action on various foodborne pathogenic bacteria, including *Escherichia coli*, a well-known etiologic agent of severe diarrhea [77]. Regarding antiviral properties, SGs seem to impede binding of rotavirus to host cells [78]. Rotavirus is commonly associated with pediatric gastroenteritis.

### 3.7. Effect on Gut Microbiota

About 1014 bacteria colonize the human digestive tract. Evidence supports the critical role of gut microbiota composition in immune-system maturation, neurophysiology, and maintenance of human health. Gut microbiota dysbiosis may cause gastrointestinal problems, allergies, obesity, cardiovascular disorders, and CNS diseases. Therefore, it can be inferred that each condition could possess a unique gut microbiota pattern and potential usefulness of these gut microbiota as potential biological markers and drug targets [79].

It has been reported that sweeteners may alter a gut microbiota population. Saccharin and sucralose modify it with no health-related consequences. In addition, acesulfame-K decreases *Akkermansia muciniphila* and promotes *Firmicutes* propagation. Since SGs are metabolized through gut microbiota, it has been suggested that they may modify the gut microbial community [80]. Nevertheless, in vitro and in vivo studies have shown no influence of SGs on gut microbiota growth [81].

## 4. Safety Aspects

For centuries, the Guaraní natives from Paraguay have used stevia without showing any side effects. Its use as a sweetener has been granted in America and Asia. However, its approval as a sweetener in Europe was delayed due to a need for additional scientific evidence supporting its safety, mainly regarding steviol. In vitro studies reported a carcinogenic effect of steviol due to a lack of DNA repair capabilities that use a particular bacterium (*Salmonella typhimurium* TM 677). Although oral steviol-glycoside doses of up to 1880 mg/kg for four weeks have displayed slight increases in oxidative damage and chromosomal aberration frequency in mice [82], the genotoxic effects of steviol have not been found in in vivo tests of up to a dosage of 8000 mg/kg [23]. Since these are extremely high doses, it is necessary to determine the SG therapeutic index in healthy volunteers to establish dosage limits for potential pharmacological application of steviol glycosides.

After the injection of steviol or stevioside into fertile broiler eggs, there was no evidence of increased embryonic mortality or structural malformations [83]. From additional studies in animal models, stevioside did not affect fertility, pregnancy, or embryonic/fetal development [84,85]. Likewise, scientific evidence supports the absence of carcinogenic activity of steviol glycosides in humans [86].

Additionally, in vivo assays have demonstrated that stevioside is converted into steviol through colon bacterial activity but then quickly transformed into steviol glucuronide, a nontoxic metabolite excreted via urine. Moreover, in chronic studies, when stevioside was added to chickens’ diets (667 mg/kg of food), there was no observed influence on growth curves. These results suggest that stevioside does not interfere with uptake of essential elements [83]. 

In 2006, after analysis of several studies carried out on stevia and steviol glycosides in humans and animals, the World Health Organization (WHO) stated: ‘stevioside and rebaudioside A are not genotoxic in vitro or in vivo and that the genotoxicity of steviol and some of its oxidative derivatives in vitro is not expressed in vivo’ [87]. Likewise, the EFSA Panel on Food Additives and Flavourings agreed on the safety of glycosylated steviol glycosides [88]. Furthermore, no food allergy related to stevia consumption as a sweetener has been reported since 2008, when high-purity SGs were introduced to the market [89]. 

Thus, safety assessment for human consumption has been based on individual evaluations of SGs (mainly stevioside and rebaudioside A) and their mutual metabolite, steviol. To date, additional SGs have been identified. Nevertheless, their specific regulatory evaluation remains still pendent. Interestingly, when rebaudiosides A, B, C, D, E, F, and M and steviolbioside were incubated with human fecal homogenates in vitro, all SGs were metabolized to steviol within 24 h. Therefore, it is suggested that for bustling regulatory processes, previous safety evaluation data obtained from individual SGs may be applied to all of the other steviol glycosides from stevia based on their common metabolic pathway [90]. 

Due to the potential cytotoxic action of steviol, reported in vitro, it has been suggested not to use it as a sweetener [91]. SGs have been extensively investigated at the preclinical and clinical level, and they display a better security profile. Remarkably, steviol is the common SG final metabolite, and although it is absorbed at the intestinal level, it is promptly excreted via urine [14]. 

## 5. Conclusions and Future Directions

Regarding use as a sweetener, it has been demonstrated that oral consumption of stevioside at recommended doses of 4 mg/kg is not teratogenic or cancerogenic. Neither stevioside nor rebaudioside A is absorbed in the human gastrointestinal tract. Both of these SGs are converted to free steviol through microbial action. Steviol is absorbed but rapidly eliminated in urine in the form of steviol glucuronide. These pharmacokinetic aspects constitute a significant advantage in terms of potential toxicity, since the more quickly a metabolite is eliminated, the more the probability of side effects occurring is significantly reduced. Thus, stevioside can be considered a safe, noncaloric, noncariogenic, nonallergenic, and natural alternative to sucrose. Although neither the applicability of stevioside in children, pregnancy, nor lactation has been evaluated, preclinical and clinical evidence of its safety allows it to be recommended, respecting the ADI. Perhaps the main limitation of its use is its bitter aftertaste. However, nowadays, enzymatic glycosylation improves sensory properties and acceptability.

Current evidence has pointed out that stevioside and rebaudioside A, administered at higher doses than ADI, can be proposed as potential drug candidates for treating cardiovascular disorders, diabetes, cancer, inflammation, diarrhea, and oxidative processes. Noteworthily, the pharmacological properties of SG are specifically observed in pathological situations. For example, stevioside does not cause hypoglycemia at normal glucose concentrations, but stevia does so. This fact highlights the need to continue investigating and characterizing the bioactivity of isolated SGs, since in stevia extract, a great variety and number of constituents can be found that could generate synergistic or inhibitory effects among themselves. Therefore, further clinical studies are needed to determine proper dosages for pharmacological purposes. 

## Data Availability

Not applicable.
